# Topographical anatomy of the mandibular foramen in the dromedary camels (*Camelus dromedarius*): an extraoral approach for the inferior alveolar nerve blocks

**DOI:** 10.3389/fvets.2023.1303810

**Published:** 2023-12-06

**Authors:** Zakriya Al Mohamad, Mohamed Hamed, Ahmed Abdellatif, Haitham Eldoumani, Elsayed Elshafaey, Khaled Abouelnasr

**Affiliations:** ^1^Department of Clinical Sciences, College of Veterinary Medicine, King Faisal University, Al Ahsa, Saudi Arabia; ^2^Department of Surgery, Anesthesiology and Radiology, Faculty of Veterinary Medicine, Aswan University, Aswan, Egypt; ^3^Department of Anatomy and Embryology, Faculty of Veterinary Medicine, Mansoura University, Mansoura, Egypt; ^4^Faculty of Veterinary Medicine, Mansoura University, Mansoura, Egypt; ^5^Department of Surgery, Anesthesiology and Radiology, Faculty of Veterinary Medicine, Mansoura University, Mansoura, Egypt; ^6^Department of Veterinary Surgery, Salam Veterinary Group, Buraydah, Saudi Arabia

**Keywords:** mandibular foramen (MF), regional anesthesia, regional anatomy, cadaveric, mandibular canal, inferior alveolar nerve (IAN)

## Abstract

Understanding the clinical anatomy of the head is essential for performing proper inferior alveolar nerve (IAN) block anesthesia to facilitate invasive dental procedures in camels. However, osteometric data related to the IAN in camels are lacking. This study was carried out to accurately locate the mandibular foramen (MF) and the course of the IAN in the camel head and to establish an approach for its localization in clinical practice. To achieve these aims, eight osteometric measurements were used to determine the location of the MF in relation to its surrounding structures in six cadaveric skulls of adult camels. Four camel heads were dissected, and the course of the IAN inside the mandibular canal was studied. In addition, four heads were used as a trial for the extraoral approach to the IAN block using contrast radiographs based on established metric indices. Dissection of the four camel heads revealed that the MF was located near the intersection of two lines passing through the occlusal surface of the mandibular cheek teeth and at the midpoint of the zygomatic process of the temporal bone. Significant differences were not observed between the right and left mandibles. Successful deposition of the contrast medium near the MF was observed in all examined specimens. This study reports a new, simple approach to reaching the IAN at the MF. However, further clinical validation of the proposed technique is required.

## 1 Introduction

The dromedary camel is a large even-toed ungulate in the tropics. Camels are well adapted to desert life, with the largest populations distributed in North Africa and West and Central Asia (1). Camel-derived products have recently demonstrated emerging therapeutic benefits (2, 3). Therefore, the interest in understanding their anatomy and physiology is increasing.

General anesthesia is considered a humane method for controlling pain during major surgical procedures in farm animals. However, this technique is associated with higher risks in ruminants than in monogastrics (4). Regional nerve block anesthesia is a reliable and cost-effective alternative and remains the most desirable technique in many surgical interventions (5). This technique involves the deposition of an anesthetic agent close to the main nerve supplying the desired region, resulting in a reversible loss of sensation. Unlike horses, in which the parameters for regional anesthesia are well established, available data related to head nerve block in other domestic animals, including camels, are scarce (6, 7). Morphological differences in craniofacial bones among domestic animals further confirm the relevance of establishing species-specific parameters as prerequisites for successful regional anesthetic techniques. Employing incorrect parameters can result in incomplete loss of sensation and/or nerve damage (8).

Understanding the regional anatomy of the head is useful for localizing various structures, such as the cranial nerves, that are relevant to clinical practice by surgeons and practitioners. The mandibular foramen (MF) is an opening located on the medial surface of the ramus of the mandible, where it forms the starting point for the mandibular canal (9). The mandibular canal contains the inferior alveolar nerve and its associated vessels, which terminate obliquely and rostrally to the mental foramina, located on the lateral surface of the mandible. The inferior alveolar nerve is a pure sensory nerve and a terminal branch of the mandibular branch of the trigeminal nerve (10). During its course, through the mandibular canal, the inferior alveolar nerve gives off branches to the lower dental arch, gingiva, skin, and mucosa of the lower lip and chin. Thus, anesthesia for this nerve is an invaluable tool for desensitizing these structures during surgery.

Several approaches have been developed for inferior alveolar nerve block in domestic animals. These approaches mainly focus on horses and can be performed via either the oral or extraoral route (11, 12). Contrast radiography has been used to assess the precise injection of local contrast infusion for several dental blocks in human and veterinary practice (6, 13). Additionally, osteometric measurements for localizing various craniofacial structures have been reported for the dried skulls of several domestic animals, including sheep (14), goats (15, 16), cows (17), donkeys (18), pigs (19), and camels (20, 21). These studies agree that species-specific differences exist in skull morphometry. Particularly, in camels, radiographic (22, 23) and gross anatomic (24–26) descriptions of the head have also been reported. The aforementioned studies suggest peculiar morphologic characteristics for the skulls of adult camels and conclude the existence of significant differences from those of ruminants and equines. However, we did not find sufficient data to provide comprehensive information on the location of the MF and the course of the IAN in camels. This study aimed to focus on the detailed clinical anatomy of the MF in camels and to establish an approach for its localization based on fixed anatomical landmarks.

## 2 Materials and methods

### 2.1 Animals

Fourteen heads [7 males (M); 7 females (F)] of adult one-humped camels (10 ± 5.7 years old) were collected from a local slaughterhouse in Al-Sharqia Governorate (Egypt) for localization of the mandibular foramen. The heads were separated from the body at the level of the atlanto-occipital joint, and age was estimated using dentition (27). All heads were collected immediately after slaughter, frozen for 1 day before the study, and then thawed. Six heads (3M; 3F) were prepared for osteometric measurements as previously described (28, 29). Four heads (2M; 2F) were dissected to study the regional anatomy. The remaining four heads (2M; 2F) were used for the localization of the IAN in the injected radiographs based on the established metric indices. The Faculty of Veterinary Medicine, Mansoura University animal welfare and use committee (No. VM.R.22.12.38) approved the study.

### 2.2 Osteometry

**Figure 1:**
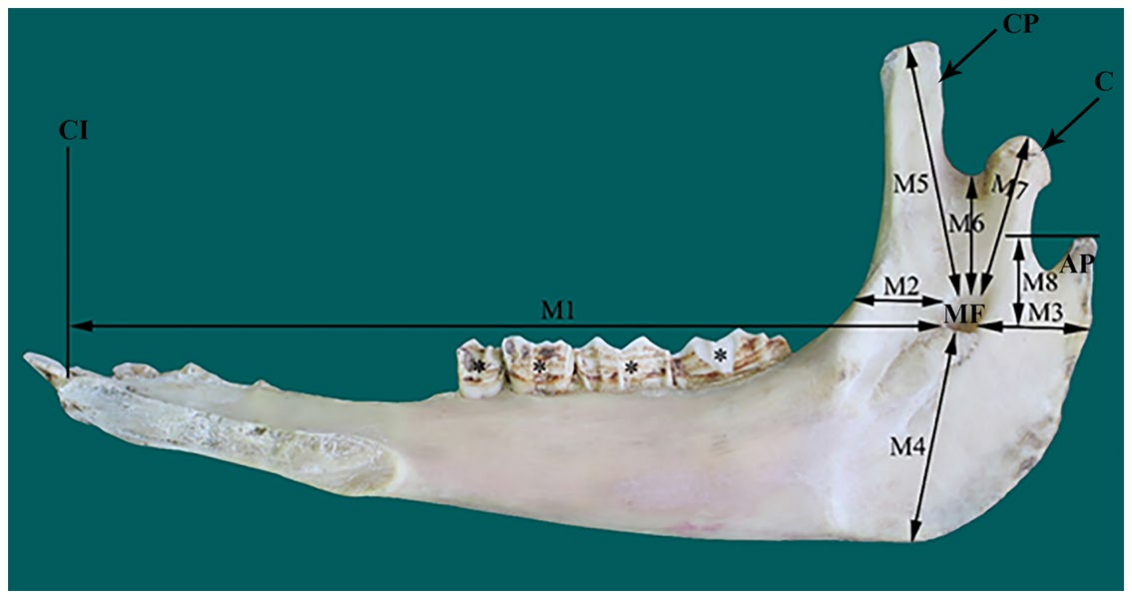
Photograph of a right mandible of adult camel (medial view) illustrating the eight osteometric measurements (M1–M8) used for localization of the mandibular foramen (MF). AP, angular process; C, condylar process; CI, root of first incisors; CP, coronoid process.

The location of MF in the dried camel skulls (12 mandibles) was determined based on eight osteometric measurements that connected the foramen with its surrounding bony landmarks (Figure 1). These measurements were recorded with a digital Vernier caliper and included distances (in cm) from the MF to the first incisor teeth (M1), the rostral border of the ramus of the mandible (M2), the caudal border of the ramus of the mandible (M3), the ventral border of the mandible (M4), the highest point of the coronoid process (M5), the mandibular notch (M6), the condylar process (M7), and the highest point of the angular process (M8).

### 2.3 Head dissection

Four camel heads were dissected and the course of the IAN inside the mandibular canal was studied. The external bony coverage of the canal was removed using a bone cutter. High-quality images were captured using a digital camera (Canon EOS 700D) and were used as a reference for subsequent studies.

### 2.4 Nerve localization in contrast radiographs

**Figure 2:**
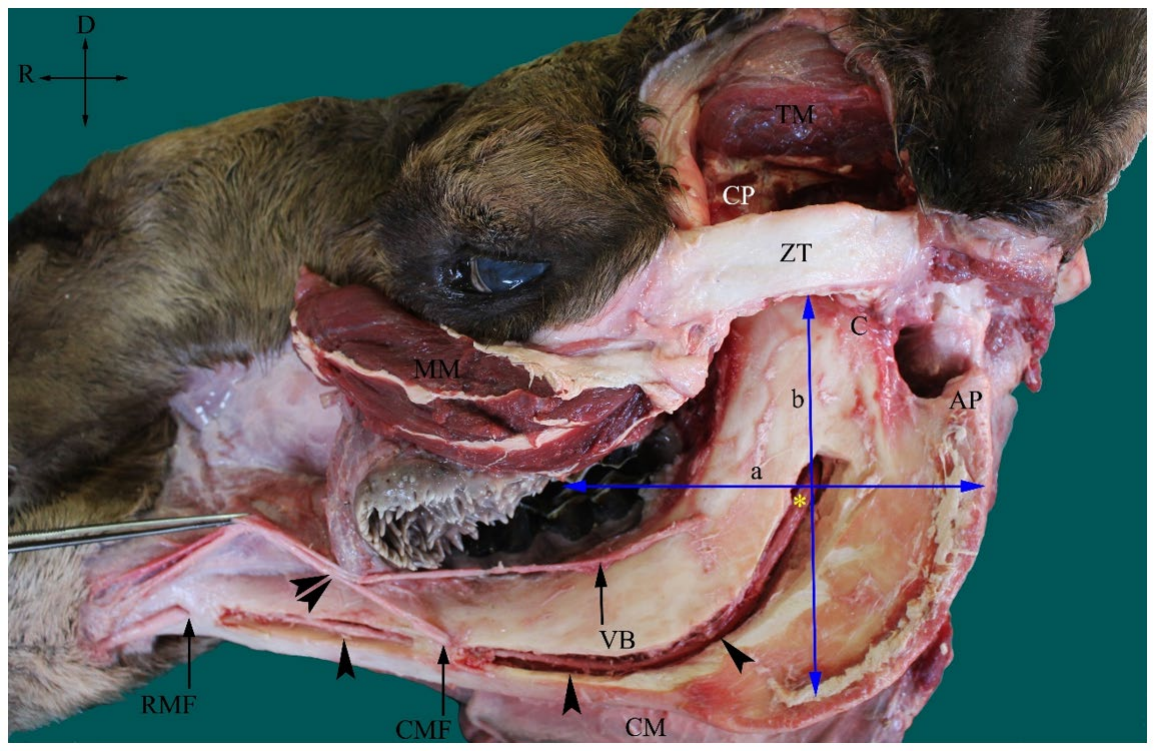
Photograph of dissected left side of an adult camel head showing the inferior alveolar nerve (IAN) (asterisk) and its course inside the mandibular canal (arrowheads) and the scheme used for its localization at the MF. The MF is located approximately at the point of intersection of the line passing at the occlusal surface of the cheek teeth (a) and the line falling from the midpoint of the zygomatic process of temporal bone (ZT). AP, angular process; C, condylar process; CM, cutaneous muscle; CMF, caudal mental foramen; CP, coronoid process; MM, masseter muscle (partially cut and reflected); TM, temporal muscle (partially removed); RMF, rostral mental foramen and VB, ventral buccal nerve. A connection between the mental nerve (following its exit from the CMF) and the VB was observed (double arrowheads). Directions are dorsal (D) and rostral (R).

The approximate site of the MF was determined based on both recorded osteometric measurements and the newly developed scheme observed in the present study. In this scheme, the MF was located approximately at the point of intersection of a line passing through the occlusal surface of the cheek teeth and another line falling from the midpoint of the zygomatic process of the temporal bone (Figure 2). Each cadaver head was positioned in ventral recumbency to mimic the head position during a standard dental examination. Contrast radiography was performed as previously described (30). To localize the MF on contrast radiographs, a 40 mm 16-gauge hypodermic needle was used to inject 5 mL of radiopaque opaminol contrast medium at the proposed site of the MF. The needle was inserted at an angle of approximately 30° from the caudal edge of the mandible along the medial aspect at a fixed distance from the highest point of the angular process until the rostral edge of the ramus was encountered. The needle point was then retracted slightly from the medial side of the ramus, allowing it to remain near the mandibular foramen. This process was repeated for all the four camel heads. Immediately after injection, radiographic images were captured using a radiography unit (Samsung-dong, SY-31-100-P, Seoul, Republic of Korea) at focal film distances of 70 kVp, 2.0 mAs, and 70 cm. Radiographs were used to evaluate the needle tip positioning and contrast diffusion patterns. The success of the injections was indicated by the presence of a contrast agent at the entrance of the mandibular canal on radiographs.

### 2.5 Statistical analysis

Statistical analyses were performed using GraphPad Prism 7.0 (GraphPad Software, San Diego, CA, United States). Differences between the right and left sides of the head were determined using student’s *t*-test. Statistical significance was set at *p* < 0.05.

## 3 Results

The MF was located on the medial surface of the vertical part of the ramus of the camel mandible, with its opening directed upward. The foramen was seated in the middle third of the horizontal part of the ramus of the mandible, slightly closer to the rostral border of the mandible than its caudal edge (Figure 1). The mental foramen was represented by a pair of foramina in the camel on the lateral side of the horizontal part of the ramus of the mandible, with the rostral foramen being wider, consistent with the larger size of the transmitted nerve (Figure 2).

**Figure 3:**
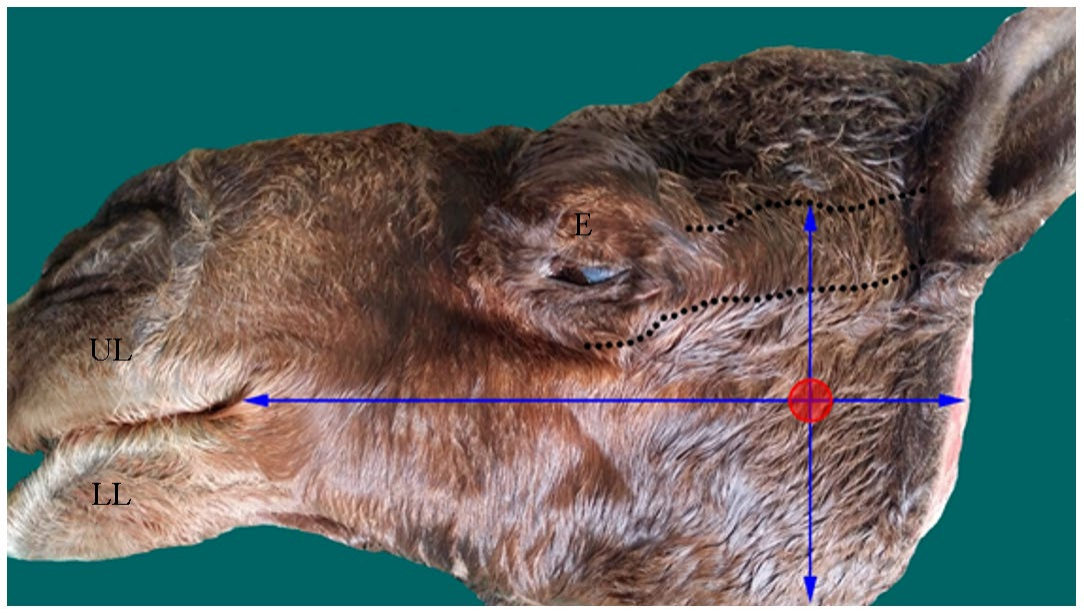
Photograph of head of an adult camel showing the approximate site (red circle) of the MF based on the established scheme. The margins of the zygomatic process of temporal bone is marked by dotted lines. E, eye; LL, lower lip; UL, upper lip.

With the osteometric measurements specified in the present study, significant differences were not observed between the right and left sides of the heads of the tested animals. Except for M2, minimal variation (coefficient of variation, <15%) was observed among the examined animals (Table 1). The distance from the MF to the caudal border of the mandible revealed little variation in dried skulls of camels 6.8% for the right side (average 5.51 ± 0.37 cm); 7.1% for the left side (average 5.42 ± 0.39 cm). The angular process of the camel has a high location in the caudal aspect of the condylar process. The distance from the MF to the highest point of the angular process was found to be 3.53 ± 0.28 cm in the right side and 3.74 ± 0.27 in the left side. After the latter distance was combined with the distance from the MF to the caudal border of the mandibular ramus, precise localization of the MF was achieved. In addition to these metric indices, dissection of the camel heads indicated that the MF was located at the point of intersection of two lines: one running horizontally at the occlusal surface of the cheek teeth and the other falling from the middle of the zygomatic process of the temporal bone towards the ventral border of the mandible (Figure 2). These landmarks were easily detectable in cadaveric camel heads and thus provided a practical way to localize the IAN upon its entrance to the MF (Figure 3). Descriptive details of the osteometric measurements used for the localization of the MF in the skull of dromedary camels are reported in Table 1.

**Table 1:** Summary for mean and standard deviation (M ± SD) of osteometric measurements used for localization of the MF in skull of dromedary camel.

Parameter	Right mandibles		Left mandibles		*p*-value
	M (cm)	SD	M (cm)	SD	
M1	28.24	1.55	28.10	1.35	0.88
M2	4.26	0.75	3.92	0.65	0.46
M3	5.51	0.37	5.42	0.39	0.73
M4	8.11	0.66	7.98	0.73	0.78
M5	9.20	0.66	9.11	0.76	0.84
M6	4.63	0.53	4.71	0.50	0.81
M7	6.50	0.75	6.71	0.93	0.70
M8	3.53	0.28	3.74	0.27	0.25

Estimated osteometric parameters are distances from the mandibular foramen (MF) to the first incisor teeth (M1), the rostral border of the vertical ramus of the mandible (M2), the caudal border of the ramus of the mandible (M3), the ventral border of the mandible (M4), the highest point of the coronoid process (M5), the mandibular notch (M6), the condylar process (M7), and the highest point of the angular process (M8).

**Figure 4:**
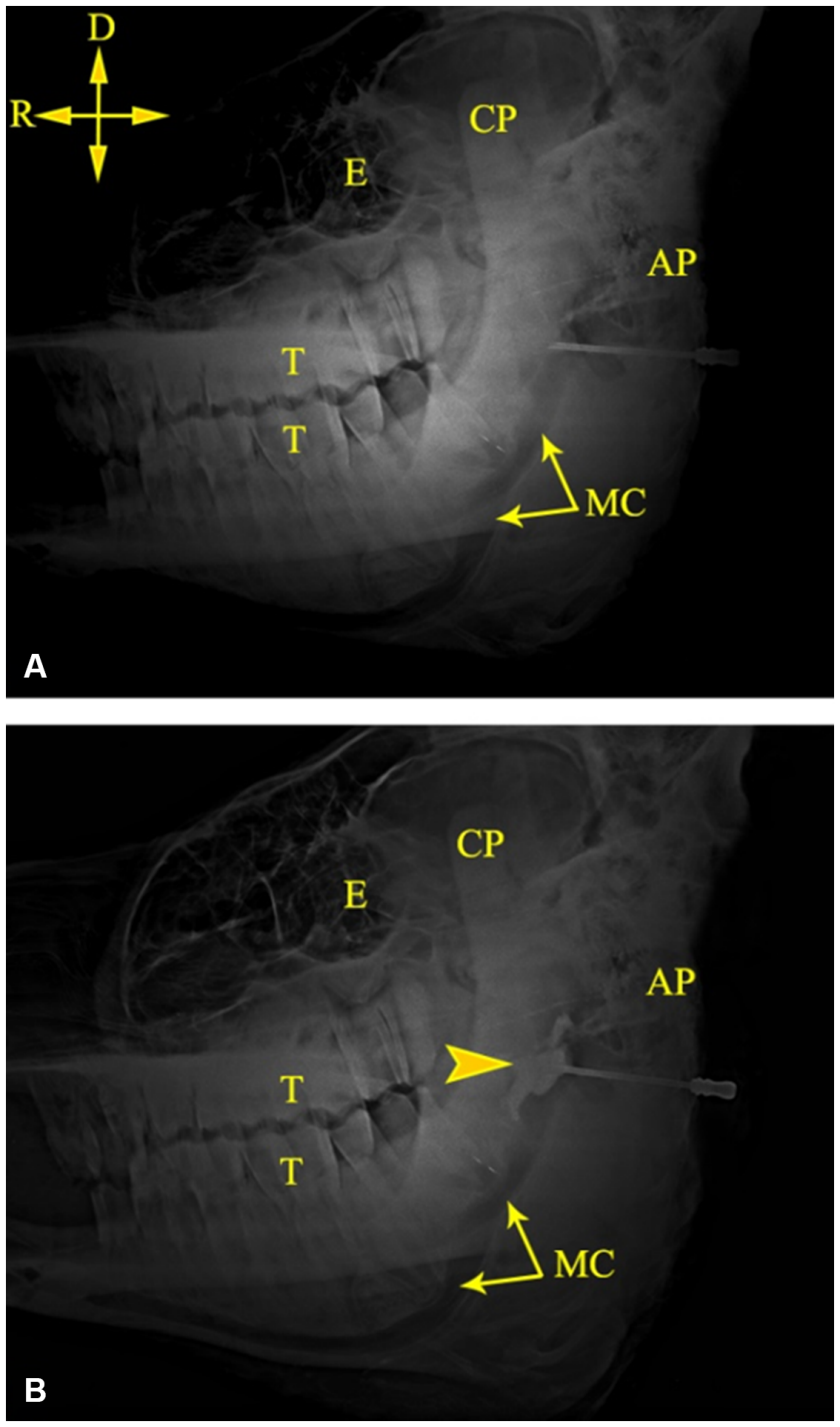
Lateral radiographs of the left side of a camel head showing the tip of the needle at the MF **(A)** and successful deposition of the contrast medium at the MF **(B)**. AP, angular process; CP, coronoid process; E, Eye; MC, mandibular canal; T, cheek teeth. Directions are dorsal (D) and rostral (R).

The mandibular canal could be easily recognized on radiographic images of camel heads owing to its radiolucent tubular appearance along the ventral border of the mandible (Figure 4). Combining measurements M3 (from the MF to the caudal border of the mandible) and M8 (from the MF to the highest point of the angular process) with the abovementioned scheme achieved perfect localization of the MF on radiographs of the injected heads, where the injected contrast agent was observed at the entrance of the canal soon after its injection (Figure 4). Additionally, radiographic imaging of the cadaver heads revealed successful placement of the contrast agent at the entrance of the mandibular foramen in all tested specimens. Needle placement at the level of the MF was easily recognized, and the contrast agent adequately infiltrated the area around the mandibular nerve.

## 4 Discussion

In this study, the distance from the MF to the caudal border of the mandible revealed little variation in the dried skulls of camels [6.8% for the right side (average 5.51 ± 0.37 cm); 7.1% for the left side (average 5.42 ± 0.39 cm)]. This distance was reported to be 5.88 ± 0.055 cm in Indian one-humped camels (20). Comparable measurements were also reported in Nigerian camels (21). Collectively, these data indicate a conserved location of the foramen in camels even with differences in their geographic locations. Moreover, the extraoral approach was accessed with a 40 mm needle inserted caudally from the highest point of the angular process until the rostral edge of the ramus was encountered on the medial side of the mandibular ramus. These differences were not detected in camels in this study or in the study by Yahaya et al. (21). Sexual dimorphism in human facial bones was caused by testosterone-associated-genetic variants (31, 32). The lack of sexual dimorphism seen in camel mandible could suggest a modest effect of testosterone on skull morphometry.

The angular process of the mandible is a peculiar anatomical landmark specific to several mammals, including camels, dogs, cats, and foxes (9, 33, 34). Unlike the skulls of dogs, cats, and foxes, in which the angular process is caudally directed and located near the basal border of the mandible at a level distal to the position of the MF, the angular process of the camel has a high location and is situated on the caudal aspect of the condylar process. We examined the position of the MF in relation to the angular process of the camel skull. Therefore, the distance from the MF to the highest point of the angular process was investigated in this study. This distance was found to be 3.53 ± 0.28 cm on the right side and 3.74 ± 0.27 cm on the left side. When the latter distance was combined with the distance from the MF to the caudal border of the mandibular ramus, precise localization of the MF was achieved.

The extraoral approach to the inferior nerve block requires a good understanding of camel anatomy. In the present study, the MF was positioned within the middle third of the medial surface of the ramus of the mandible, which is in line with the occlusal surface of the lower cheek teeth (Figure 1). In the dissected heads, the foramen appeared distal to the midpoint of the zygomatic process in the temporal bone (Figure 2). As these two criteria were consistent in all the examined heads in the present study, a scheme for localizing the MF was established based on them. A similar scheme for localizing MF in horse heads was used by Harding et al. (11). The latter approach was based on locating the MF at the intersection of two imaginary lines passing through the occlusal surface of the lower cheek teeth, and the lateral canthus of the eye. The variation in our reported scheme for camel heads and that used for MF localization in horses could be explained by differences in the anatomical locations of the orbit in relation to the MF in the two species. The camel orbit is more rostral than the horse orbit in relation to the ramus of the mandible because of the presence of a well-developed zygomatic process in the temporal bone in the former species.

The IAN block is a well-established technique in human dentistry. This technique was mostly performed using an intraoral approach based on the known anatomy of the region (35). The extended shape of the camel’s head and the relatively long distance from the root of the incisor teeth to the MF (28.24 ± 1.55 for the right side; 28.10 ± 1.35 for the left side) observed by the present study indicate difficulties in locating the MF using an intraoral approach. Alternatively, a safe extraoral approach for locating the MF in camels was indicated in this study. Radiographic imaging of the cadaver heads revealed successful placement of the contrast agent at the entrance of the mandibular foramen in all tested specimens. Needle placement at the level of the MF was easily recognized, and the contrast agent adequately infiltrated the area around the mandibular nerve. Accurate placement of the needle tip relative to the MF requires a small volume of the anesthetic agent (5 mL). A larger volume may increase the risk of neural ischemia secondary to increased regional pressure and allow diffusion of the injected agent beyond the target anatomical site. However, the present study was carried out in cadavers and thus did not consider animal compliance or local blood flow in the sedated camel.

The limitations of this study include the small number of cadaveric specimens used. Application of the IAN block to cadaveric cases excluded vital *in vivo* parameters such as pain, circulation, and animal movement during injection. In addition, the viscosity of the contrast agent may be different from that of the local anesthetic and may influence injection pressure and diffusion.

## 5 Conclusion

Nerve block anesthesia is often required in conjunction with sedation to facilitate invasive procedures in dental surgery. The present study reports new findings related to the position of the MF in camels and possible implications for regional anesthesia of the IAN. Furthermore, a simple osteometry-and regional anatomy-based approach for desensitizing the IAN was discussed in light of these findings. However, several larger optimizations are required to validate the proposed approach in clinical practice using the correct dose, type of anesthetic agent, and appropriate needle size.

## Data availability statement

The raw data supporting the conclusions of this article will be made available by the authors, without undue reservation.

## Ethics statement

The animal studies were approved by the Institutional Animal Care and Use Committee of Faculty of Veterinary Medicine, Mansoura University (Approval No. VM.R.22.12.38). The studies were conducted in accordance with the local legislation and institutional requirements. Written informed consent was obtained from the owners for the participation of their animals in this study.

## Author contributions

ZM: Resources, Software, Validation, Writing – review & editing. MH: Data curation, Investigation, Methodology, Writing – review & editing. AA: Conceptualization, Formal analysis, Validation, Writing – original draft, Writing – review & editing. HE: Software, Writing – review & editing. EE: Conceptualization, Data curation, Investigation, Validation, Writing – original draft, Writing – review & editing. KA: Formal analysis, Methodology, Writing – review & editing.
